# Cervical Ribs

**Published:** 1914-02

**Authors:** 


CERVICAL RIBS. *Soc. Med. des Hop.*, July 11, 1913, reports four cases. In one there was Raynaud's disease, in two Claude Bernard's syndrome, in one hereditary syphilis which leads to the suggestion that the formation of extra ribs may be a syphilitic dystrophy.

